# Confusion Syndrome During COVID-19: The “Herb” That Hides the Forest?

**DOI:** 10.7759/cureus.17775

**Published:** 2021-09-06

**Authors:** Asmaa Hazim, Fadil Bakkali, Sara Lhassani, Karim El Aidaoui

**Affiliations:** 1 Neurology, Mohamed VI University of Health Sciences, Faculty of Medicine, Cheikh Khalifa Ibn Zayed Hospital, Casablanca, MAR; 2 Toxicology Laboratory, Mohamed VI University of Health Sciences, Faculty of Medicine, Cheikh Khalifa Ibn Zayed Hospital, Casablanca, MAR; 3 Intensive Care Unit, Mohammed VI University of Health Sciences, Cheikh Khalifa Ibn Zayed Hospital, Casablanca, MAR

**Keywords:** covid-19, confusion, delirium, neurology, dysphania ambrosioides, toxic encephalopathy.

## Abstract

We report the case of potential *Dysphania ambrosioides *(Silverweed) intoxication in the context of SARS-COV-2 infection in a patient admitted for delirium with Glasgow Coma Score (GCS) of 13/15. This herb was used as an antipyretic to treat COVID-19 persistent fever. The clinical presentation of our patient raised several questions related to the viral or herbal intoxication origin of the confusion syndrome. To our knowledge, this is the first description of toxic encephalopathy after *D. ambrosioides *ingestionin an adult patient.

## Introduction

Several neurological manifestations associated with COVID-19 have been reported during this global pandemic. The neuro-invasive potential of the SARS-COV-2 has been described and many case series reported frequent mental confusion especially in intensive care units. Nevertheless, clinicians should not forget to deeply explore the other possible origins of a confusion syndrome. 

## Case presentation

A 39-year-old patient with a history of treated pulmonary tuberculosis followed up initially at home before hospital admission for SARS-COV-2 infection revealed by fever, anosmia, and ageusia. COVID-19 real-time reverse transcriptase-polymerase chain reaction (RT-PCR) test on nasopharyngeal swab was positive. On day 5 of infection, the patient was admitted to the hospital for delirium.

The initial clinical examination reported a confused patient with temporospatial disorientation, inconsistent speech, and a GCS of 13/15. His central temperature was at 37.5 °C. The patient’s blood pressure was normal (130/90 mmHg) with a heart rate of 90 bpm and peripheral capillary oxygen saturation of 95%. The clinical examination highlighted a cutaneous maculopapular and erythematous rash affecting the face and the trunk. The neurologic examination was normal.

Interestingly, the family interview revealed the use of unspecified amounts of *Dysphania ambrosioides *- based oral infusions for five days to treat his fever. The brain MRI was unremarkable. Cerebrospinal fluid analysis (CSF) revealed a clear liquid (rock water) with negative direct examination, leukocytes <5 elements /mm^3^; proteinorachia at 0.45 g/l, and glycorachia at 0.63 g/l. 

Laboratory tests and hematological parameters were as follows: sodium at 132 mmol/l, C-Reactive Protein at 20 mg/l (normal range < 8), neutrophilic leukocytosis at 11.103/mm^3^ without lymphopenia, ferritin concentration at 200 ng/ml (normal range 30-300). Coagulation tests, as well as infectious serologies, were negative. Renal and hepatic functions were strictly normal as well as tests for toxins in the urine (alcohol, amphetamine, methamphetamine, barbiturates, benzodiazepine, cocaine metabolites, and opiates) which returned negative. Vitamin B1, B6, B9, and B12 dosages were within the normal range. 

**Figure 1:**
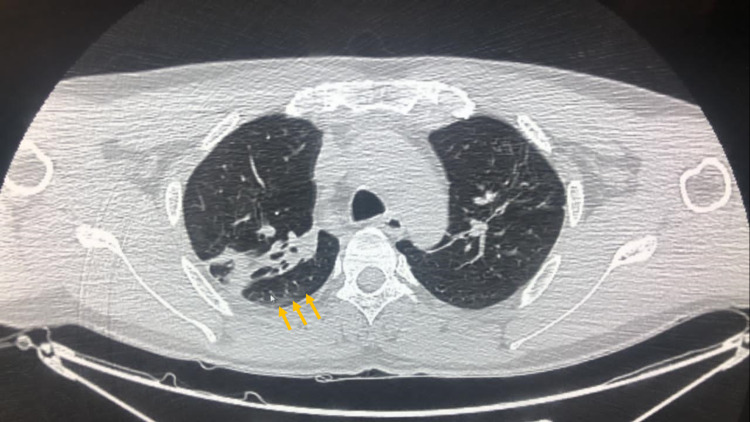
Chest CT-Scan Chest CT-Scan showing asymmetric bilateral ground-glass opacities estimated between 5-10% (yellow arrows)

**Figure 2:**
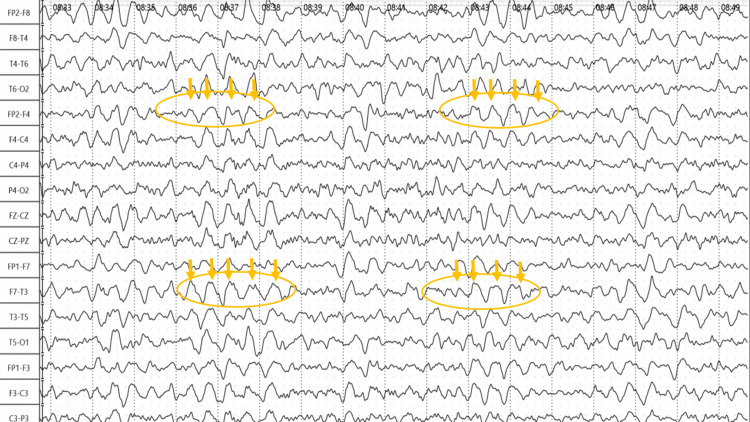
EEG EEG tracing showing confusion with slow waves in the anterior region (yellow arrows)

The chest CT-Scan showed asymmetric bilateral ground-glass opacities estimated between 5-10% (Figure 1) and the electroencephalogram (EEG) showed a tracing of confusion with slow waves in the anterior regions (Figure 2).

A probabilistic antibiotic and antiviral treatment (based on Ceftriaxone 6g/day and Acyclovir 10mg/kg/day) was started in the emergency department and stopped upon receipt of the results of CSF analysis. 

The latter's evolution was marked by the regression of the delirium 24 h after the admission as well as the cutaneous rash. The patient was discharged 48 hours after admission and seen one week later in ambulatory care where his neurological examinations were unremarkable.

## Discussion

The clinical presentation of our patient raised several questions related to the viral or herbal intoxication origin of the confusion syndrome. Indeed, in the neurological manifestations associated with COVID-19, the direct damage to the central nervous system by the SARS-COV 2 does not appear to be in the foreground. Nevertheless, the neuro-invasive potential of the virus exists and has been described during this pandemic. Rogers et al. mention frequent mental confusion reaching 65% of patients in intensive care [1]. Meppiel et al. reported that disturbances of consciousness were observed in 78% of encephalopathy cases in their series [2]. However, consciousness disorders tend to be significantly more frequent in severe forms of COVID-19 than non-severe cases (14.8% vs. 2.4%, in Mao's study) [3]; their mechanism is not unequivocal: direct attack on the parenchyma by the virus, epileptic seizures, toxic/metabolic encephalopathy. These toxic or metabolic mechanisms can be of multiple origins (e.g. cytokine storm, sepsis, and its inflammatory procession, renal failure or ionic disorders) [2,4,5,6]. In these cases, there are no specific abnormalities both on brain MRI and CSF examination [5]. 

The cases of meningoencephalitis directly associated with SARS-CoV-2 are rather rare, ranging from 6% to 9.5% depending on the series [2-7]. However, in a large French study including 222 patients with neurological disorders, on 97 CSF examinations, pleocytosis was only present in 18 patients (18.6%) and RT-PCR for SARS-CoV-2 was positive only in 2.1% of the explored cases [2]. A para-infectious mechanism seems to be frequently involved, especially when encephalitis apparition is often delayed (up to 17 days after the onset of infectious signs) and the response to corticosteroids (when it has been tried) was very good [2,8]. 

On the other hand, *Dysphania ambrosioides *is an annual or perennial shrub with a strong aromatic odor cultivated in the subtropical and sub temperate regions around the world [9-10]. In folk medicine, all parts (roots, leaves, flowers, bark, seeds) of *D. ambosioides* are used as poultice, for baths, oral ingestion or as tea to treat influenza and other respiratory diseases as well as digestive, urogenital, vascular, and nervous disorders [11-14]. The main components of *Dysphania ambroisoides* essential oil are α-Terpinene (7-53%), p-cymene (2-10%), ascaridole (16-61%), and ascaridole epoxide. These chemical compounds can vary according to several parameters such as climate and soil. Ascaridole is very poorly soluble in water and can concentrate in the essential oil of *D. ambrosoides*. Monzote et al. showed that the toxicity of the essential oil from *Dysphania ambrosioides* is partially related to the inhibition of the respiratory chain complex preferably by caryophyllene oxide while the toxicity of ascaridole is dependent on the availability of redox-active iron. Ascaridole was less toxic to mammalian mitochondria than other major ingredients. However, under the availability of redox-active Fe2+, reduced hemin, and oxidative stress conditions, the toxicity can potentially be increased. [15-17] 

In Morocco, *D. ambrosioides* (named locally M’khinza) is frequently used for its antipyretic, analgesic, hemostatic and antispasmodic effects. However, prolonged and uncontrolled exposure to this plant can lead to more or less significant renal, gastrointestinal, and also encephalic complications depending on the duration of exposure and the dose administered [16]. Data on its encephalic damage is scarce and *D. ambrosioides* intoxication cases are poorly documented. To our knowledge, only two cases affecting the pediatric population were reported in the literature. Clinically,* D. ambrosioides*-related toxic encephalopathy may present with mental confusion, behavioral disturbances, vigilance disorders ranging from obtundation to deep coma, myoclonus, and seizures. In all cases, clinical improvement is essential to confirm the diagnosis and given the absence of an antidote, the treatment is purely symptomatic. [18-19]

In our case, the patient presented with a mild form of COVID-19, both brain MRI and CSF were normal, his biological inflammatory parameters were moderate without ionic disorders, with neither hepatic nor kidney function disturbances. His EEG did not show any seizure abnormalities and the evolution of confusion as well as cutaneous rash was rapidly resolutive after cessation of *D. ambrosioides* ingestion and symptomatic treatment. All these arguments tend to support the herbal intoxication origin of the confusion syndrome.

## Conclusions

The case of our patient should remind clinicians, especially in the countries where traditional medicine is frequently used, to consider the possibility of medicinal plants intoxication as their prescription, preparation, and consumption are not always obvious nor automatically revealed during anamnesis.
